# Longitudinal analysis of minority women’s perceptions of cohesion: the role of cooperation, communication, and competition

**DOI:** 10.1186/1479-5868-11-57

**Published:** 2014-04-29

**Authors:** Samantha M Harden, Paul A Estabrooks, Scherezade K Mama, Rebecca E Lee

**Affiliations:** 1 Virginia Tech, Department of Human Nutrition, Foods and Exercise, Virginia Tech, Blacksburg, VA, USA; 2 University of British Columbia, School of Kinesiology, Vancouver, BC, USA; 3 Fralin Translational Obesity Research Center, and Department of Human Nutrition, Foods and Exercise, Virginia Tech, Blacksburg, VA, USA; 4 Department of Family and Community Medicine, Carilion Clinic, 1 Riverside Circle, Suite 104, Roanoke, VA 24016, USA; 5 Texas Obesity Research Center, Health and Human Performance, University of Houston, Houston, TX, USA; 6 Department of Health Disparities Research, The University of Texas M.D. Anderson Cancer Center, Houston, TX, USA; 7 College of Nursing and Health Innovation, Arizona State University, Phoenix, AZ, USA

**Keywords:** Group dynamics, Group-interaction, Physical activity

## Abstract

**Background:**

Interaction in the form of cooperation, communication, and friendly competition theoretically precede the development of group cohesion, which often precedes adherence to health promotion programs. The purpose of this manuscript was to explore longitudinal relationships among dimensions of group cohesion and group-interaction variables to inform and improve group-based strategies within programs aimed at promoting physical activity.

**Methods:**

Ethnic minority women completed a group dynamics-based physical activity promotion intervention (N = 103; 73% African American; 27% Hispanic/Latina; mage = 47.89 + 8.17 years; mBMI = 34.43+ 8.07 kg/m^2^) and assessments of group cohesion and group-interaction variables at baseline, 6 months (post-program), and 12 months (follow-up).

**Results:**

All four dimensions of group cohesion had significant (ps < 0.01) relationships with the group-interaction variables. Competition was a consistently strong predictor of cohesion, while cooperation did not demonstrate consistent patterns of prediction.

**Conclusions:**

Facilitating a sense of friendly competition may increase engagement in physical activity programs by bolstering group cohesion.

## Background

Group dynamics includes the study of the nature of groups, individual relationships within groups, and interactions with others. Over 60 years ago, Kurt Lewin’s seminal work suggested that the degree to which a group was cohesive would determine individuals’ level of success as a collective [1]. Group cohesion has a long history as an important predictor of performance and outcomes in work, military, sport, and exercise groups [2-4]. Having a strong sense of group cohesion also a reflects a fundamental human need—the need to belong [5].

Group cohesion has been defined in many ways [6-8], but Carron, Brawley, & Widmeyer’s [9] definition has been used consistently in physical activity promotion and research. They define group cohesion as a dynamic process reflected in the shared pursuit of common objectives to satisfy members’ needs [9]. Group cohesion is further operationalized as individual (1) attraction to the group’s task-based activities (ATG-T), (2) attraction to the group’s social activities (ATG-S), (3) perceptions of the group’s integration around task-based activities (GI-T), and (4) perceptions of the group’s integration around social activities (GI-S).

Over the previous two decades, a large body of literature has also documented the positive relationship between group cohesion and physical activity adoption and maintenance [10-14]. Participants who have strong perceptions of group cohesion attend group sessions more often, are late less often, and drop out less frequently [11]. Group cohesion also has demonstrated a consistent relationship with positive attitudes toward physical activity and enhanced perceptions of self-efficacy and personal control [4].

From a theoretical perspective, Carron and Spink [15] proposed that group-interaction variables, such as communication, cooperation, and competition, are the likely precursors to developing group cohesion. Communication is defined as the sharing of information through verbal and non-verbal means. In group dynamics-based physical activity interventions, task communication (i.e., physical activity-based) occurs through facilitated group-goal setting and peer-learning activities (c.f., Irish et al. [16], Estabrooks [17]). Cooperation is defined as sharing resources to achieve a specific outcome. In physical activity classes, a cooperative environment would be one where participants provide assistance to one another in setting up the exercise equipment, overcoming obstacles, or even doing exercises together [18]. Finally, competition in an exercise group context is defined as providing participants the motivation of being superior to other groups or group members, or the motivation to keep their own group functioning at a high level (c.f., Kerr et al. [19], Steiner [20]). The concept of friendly competition influencing physical activity outcomes has been applied across a number of populations [21-23]. Friendly competition is a sense of competition that is connected to the overall success of the group and can reflect a generalized sense of intragroup competition as well as intergroup competitions within a single intervention. With friendly competition, people are inspired to compete against each other with the recognition that even if someone else wins, it benefits the group as a whole. Further, a group may share a set of norms around fairness and reciprocity in the form of competition [24]. The motivational aspect of friendly competition has been identified across a number of studies including faith-based weight loss trials that include physical activity [25], worksite physical activity programs [26], and physical activity promotion in hard to reach audiences [27]. Gaining better understanding of the relationships between group communication, cooperation, and competition has both theoretical and practical implications.

Theoretically, to date there has been no study directed at understanding the relative contributions of communication, cooperation, and competition-based strategies to changes in group cohesion [10]. For example, it could be hypothesized that physical activity groups are more cooperative than competitive and that strategies focusing on cooperation may be superior in this context. Alternatively, communication that includes a focus on helping participants identify similarities in health aspirations could be a stronger predictor of group cohesion than cooperation or competition. However there is a lack of longitudinal or even cross-sectional research that has analyzed the change in perceptions of group-interaction variables as it relates to the change in cohesion over time.

From a practical perspective, group-interaction variables have been used as a guide to develop strategies that are hypothesized to improve group cohesion, yet the relationship between group interaction variables and group cohesion has not been examined within these intervention studies [18]. While the depth of research on other variables that enhance cohesion exists, these specific group-interaction variables have been widely used as a guide to develop strategies that are, in turn, hypothesized to improve group cohesion, yet the relationship between group interaction variables and group cohesion has not been determined [18]. Further, no study to date has analyzed the change in perceptions of group-interaction variables as it relates to the change in cohesion over time. Understanding the mechanistic relationship between strategies that target group-interaction variables, changes in those variables, and changes in perceptions of group cohesion provides valuable information for future program development. A longitudinal study design enhances the ability to track changes in the relationships between group-interaction variables and group cohesion over time. This is significant to group-based physical activity promotion programs, because it allows programs to be planned to integrate the strategies that are most likely to improve group cohesion. Understanding these underlying mechanisms also provides health educators with the information necessary to ensure that strategies that do not contribute to changes in perceptions of group cohesion are not unnecessarily applied in practice settings where resources are often limited.

To date, there have been no investigations examining the relationship between physical activity group cohesion and group member perceptions of communication, cooperation, and friendly competition [10]. Understanding these relationships could aid in developing stronger strategies (e.g., appropriate facilitation of friendly competition within a group) to enhance group cohesion and provide practical information for those delivering interventions when making resource allocation decisions (i.e., what resources are needed to deliver the intervention to the desired population, ranging from time to materials).

The Health is Power (HIP) trial [28], a study testing the effectiveness of a group-based physical activity promotion program for ethnic minority women, provided an opportunity to explore the relationships among the dimensions of group cohesion and communication, cooperation, and competition over time. In the primary meditational analyses of HIP, the investigators found that all dimensions of group cohesion mediated the effect of the intervention with regard to psychosocial outcomes but not physical activity behaviors (i.e., the intervention was associated with increased cohesion but did not lead to increased physical activity) [29].

The purpose of this exploratory study was to determine the longitudinal relationship of communication, cooperation, and friendly competition to the dimensions of group cohesion. Specifically, the intention of this study was to test participants’ perceptions of the strength or presence of these variables within their group. It was hypothesized that each group-interaction variable would contribute to a large proportion of explained variance in group cohesion over time.

*Hypothesis 1*: Cooperation would predict the Individual’s Attraction to the Group-Task.

*Hypothesis 2*: Friendly competition would predict the Individual’s Attraction to the Group-Task as well as the Group’s Integration towards the Task.

*Hypothesis 3*: Social communication would predict aspects related to social cohesion (both Individual’s Attraction to the Group-Socially as well as the Group’s Integration Socially).

*Hypothesis 4*: Task communication would predict aspects related to task cohesion: both the Individual’s Attraction to the Group-Task as well as the Group’s Integration towards the Task).

*Hypothesis 5:* Perceptions of group cohesion and group interaction would increase over the course of the program, but decrease from program completion to the 12-month follow-up.

**Figure 1:**
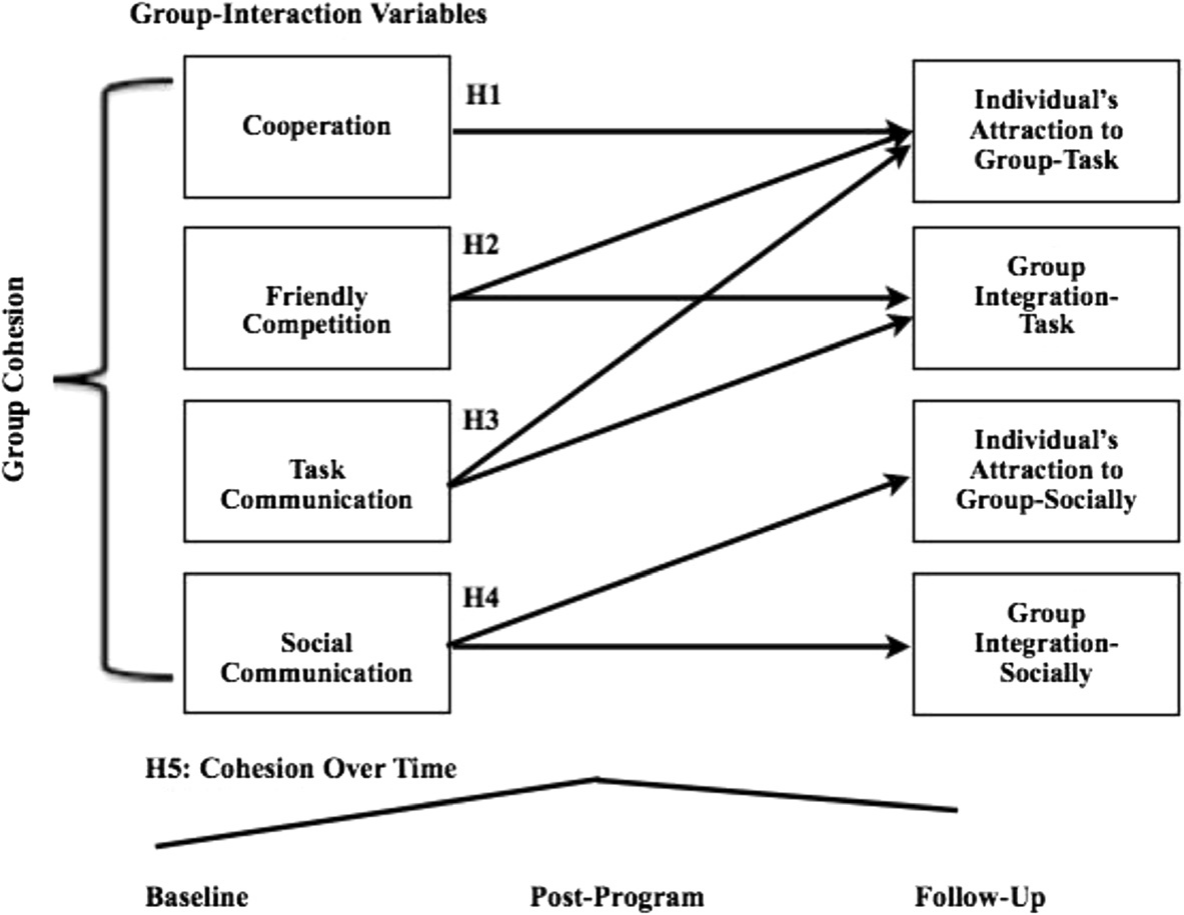
Hypotheses for group-interaction variables prediction of cohesion over-time.

Testing the hypotheses, outlined in Figure 1 below, contributes to the gap in the literature about strategies that influence the perception of group cohesion.

## Methods

African American and Hispanic or Latina women were recruited to participate in a multi-site, community-based study to test a 6-month intervention designed to promote physical activity (see Lee et al. [29] for details on recruitment). This study focused on ethnic minority women because they have particularly low rates of physical activity and disproportionately suffer from chronic diseases related to physical inactivity [30]. Further, ethnic minority women are often gatekeepers for physical activity behaviors within their families [31,32].

Women were randomly assigned to the physical activity intervention or a fruit and vegetable promotion matched contact comparison group. Only participants randomly assigned to the physical activity intervention group were included in this study. Health education intervention sessions and intervention content and materials have been previously described [28]. In Houston, there were six African-American and one Hispanic or Latina cohorts, and there were three Hispanic or Latina cohorts in Austin. All sessions were conducted in English. Intervention group sizes did not differ significantly, with a range of participants from 10 to 37.

The intervention was 24 weeks in duration and included 6 sessions. Each session included group dynamics strategies and principles based on the model developed by Carron and Spink [15] and included 15 minutes of walking after the educational component. Opportunities for communication were provided in the form of small group discussions related to the session objective. For example, women discussed strategies to overcome barriers to physical activity, shared goals, and relapse prevention plans during their group sessions. Women also used time before and after the sessions, as well as during group walks at the end of the intervention sessions, to socialize. To facilitate cooperation, participants engaged in peer problem solving activities and collaborative group goal setting. Groups also engaged in friendly competition by developing small teams working to achieve a group goal for physical activity tacked on a large map; whichever team had traveled the farthest was “winning”. The facilitators were trained to foster group interaction using semi-structured scripts. For example, a semi-structured script focusing on friendly competition might include:

“You are all part of one big team (the whole group) as well as a part of a smaller team. The small teams will be the folks that you set your shared goals with and we will have a friendly competition between each team. But I want you to remember, that the goal of our large group is that everyone in the program works their way up to 45 minutes of physical activity on at least 5 days a week. So, even though there may be some competition, this program is really about getting and giving support to be more physically active.”

Participant perceptions of physical activity group cohesion, communication, cooperation, and competition were assessed at baseline, post intervention, and 6-months after the intervention was completed. All research study activities were approved by the University of Houston’s Committee for the Protection of Human Subjects, and all participants provided written informed consent prior to participation.

### Sample

As the focus of this study was to determine the effect of group communication, cooperation, and competition on cohesion within a sample of minority women, data from the 103 participants randomly assigned to the physical activity group who completed baseline and post-intervention measures were analyzed. Of those women, 73% identified as African American and 27% identified as Hispanic or Latina. The participants were 47.89 years of age (±8.17), with an average BMI of 34.43 kg/m^2^ (±8.07). Eighty-three participants (80%) completed the 12-month follow-up assessment.

### Measures

#### Group cohesion

The Physical Activity Group Environment Questionnaire (PAGEQ) [33] is a group cohesion inventory for physical activity groups and was used in the HIP trial. The PAGEQ is a 21-item measure of the four dimensions of ATG-T, ATG-S, GI-T, GI-S with 6, 6, 5, and 4 items, respectively. All 21 items are on a 9-point Likert scale ranging from ‘strongly agree’ to ‘strongly disagree’ [33]. ATG-T was assessed by having participants respond to items such as ‘I like the amount of physical activity I get with this group’. ATG-S was operationalized through statements such as ‘I enjoy my social interactions with this group’. The group integration dimensions of cohesion were assessed using items such as ‘members of our group often socialize together’ (GI-S) and ‘our group is united in its beliefs about the benefits of regular physical activity’ (GI-T). This questionnaire has demonstrated content, predictive, and concurrent validity [33].

#### Group-interaction variables

Additional items were embedded within the PAGEQ to measure the group-interaction variables of interest, including communication, cooperation, and competition. As this was an exploratory study, we developed items that were reviewed for face validity and aligned with the definitions of each of the constructs. Like cohesion, communication was operationalized as having a task and social focus and was measured through 6 items that can be further divided into task-based communication (e.g., ‘members of our group talk about how often they should do physical activity’) and social communication (e.g., ‘people of this group talk about things that are happening in our lives’). Cooperation and competition were not conceptualized as having relevant social components and focused on task outcomes. Cooperation was measured through 3 items (e.g., ‘we all cooperate to help this group’s program run smoothly’) as was competition (e.g., ‘There is friendly competition within the members to stay as healthy as possible’). Internal consistencies for the group-interaction variables were all acceptable: task communication (α = .94), social communication (α = .65), cooperation (α = .91), and friendly competition (α = .81). The sessions were designed to enhance opportunities for each of these constructs (e.g., opportunities to cooperate before, during, and after class, facilitated social interactions) (Additional file 1).

### Analysis

Descriptive statistics, paired sample t-tests, and multiple regressions were conducted in IBM SPSS 19.0 with a priori significance set at p < 0.05. Within participant t-tests were conducted to determine changes in the group cohesion and interaction variables over time. Multiple linear regression was conducted to detect which group-interaction variables predicted group cohesion over the course of the program and at 12-months follow-up, accounting for age and ethnicity. Four regressions were completed, one for each dimension of cohesion as the dependent variable to test hypotheses 1-4, using the group interaction variables as independent variables. Longitudinal change scores (from baseline to post-intervention and post-intervention to follow-up) were computed for each group-interaction variable and dimension of cohesion for use in the regression models. Overall perceptions of cohesion were recorded at baseline, post-program, and follow-up to determine the trend in the perceptions of cohesion over time (hypothesis 5).

## Results

Table 1 includes the descriptive data across time for the group cohesion and group-interaction variables. Age and ethnicity did not contribute to a significant proportion of the variance in the models. As can be noted in the Table, all four group-interaction variables (task and social communication, cooperation, competition), as well as three dimensions of cohesion (ATG-T, ATG-S, GI-T), significantly increased from baseline to post-intervention (p < .05), and the magnitudes of the changes were moderate to large (Cohen’s d ranging from 0.5-0.89). GI-S significantly decreased from baseline to post-intervention, and the magnitude of change was moderate (Cohen’s d = 0.64). All variables had a significant decrease from post-intervention to 12-month follow-up in the range of small to moderate effect sizes (Cohen’s d ranging from 0.27-0.53).

**Table 1:** **Descriptive statistics of group-interaction variables over time**^**1**^

**Group-interaction variable**	**Baseline M**	**Post-program M**	**Follow-up M**
	**(n = 103)**	**(n = 103)**	**(n = 83)**
ATG-T	6.24 (SD ± 1.08)	7.19* (SD ± 1.37)	6.53** (SD ± 1.57)
ATG-S	6.09 (SD ± 1.30)	6.81* (SD ± 1.41)	6.25** (SD ± 1.46)
GI-T	5.64 (SD ± 1.46)	6.86* (SD ± 1.27)	6.21** (SD ± 1.48)
GI-S	5.17 (SD ± 1.27)	4.16* (SD ± 1.77)	3.92 (SD ± 1.64)
Cooperation	5.77 (SD ± 1.64)	6.94* (SD ± 1.41)	6.28** (SD ± 1.74)
Friendly competition	6.29 (SD ± 1.28)	6.97* (SD ± 1.38)	6.44** (SD ± 1.52)
Social communication	5.83 (SD ± 1.33)	6.71* (SD ± 1.56)	6.28** (SD ± 1.60)
Task communication	5.66 (SD ± 1.49)	7.00* (SD ± 1.47)	6.32** (SD ± 1.75)

^1^*Significant change (p < .05) between baseline and post-program.

**Significant change (p < .05) between post-program and follow-up.

ATG-T (Attraction to the Group’s Task); ATG-S (Attraction to the Group Socially), GI-T (Group’s Integration towards the Task); GI-S (Group’s Integration Socially).

The proportion of explained variance in ATG-T at each time point and was approximately 30 percent for each of the longitudinal regression analyses (see Table 2). Participant perceptions of competition and task-based communication were consistent contributors to the variance explained in the longitudinal regression. The group-interaction variables seemed to explain a slightly higher amount of the variance in GI-T when considering longitudinal data (i.e., approximately 63 percent of the variance). Task-based communication and friendly competition were again significant contributors to the explained variance within the longitudinal regression.

**Table 2:** Longitudinal regression results predicting each dimension of group cohesion

	**ATG-T**		**ATG-S**		**GI-T**		**GI-S**	
	**T1-T2**	**T2-T3**	**T1-T2**	**T2-T3**	**T1-T2**	**T2-T3**	**T1-T2**	**T2-T3**
R^2^	0.31	0.33	0.59	0.43	0.61	0.63	0.30	0.21
β cooperation	-0.09	0.13	0.12	0.22*	0.10	0.18*	0.07	0.08
β friendly competition	0.20*	0.31*	0.43*	0.37*	0.14*	0.23*	0.06	0.22*
β social communication	-0.16	-0.01	0.46*	0.31*	0.04	0.19*	0.25*	0.20
β task communication	0.54*	0.33*	-0.15	-0.06	0.61*	0.44*	0.26*	0.12

*(p < .05)^2^.

^2^ATG-T (Attraction to the Group’s Task); ATG-S (Attraction to the Group Socially), GI-T (Group’s Integration towards the Task); GI-S (Group’s Integration Socially).

The regression analyses used to examine ATG-S showed a significant amount of explained variance within a longitudinal (43 to 59 percent) approach. Social communication and friendly competition were significant contributors to the explained variance in the longitudinal regression of ATG-S. A somewhat lower proportion of variance was explained using the group-interaction variables with GI-S (25 percent longitudinally). There were no distinct patterns across the regressions for GI-S, which was predicted by social and task-based communication (T1-T2) and by friendly competition (T2-T3).

## Discussion

Communication, cooperation, and competition are key variables in the prediction of group cohesion [15,18]. Findings support the propositions from Carron and Spink’s model that some of these variables are significantly related to group cohesion [15]. We extended the findings of previous studies to show that friendly competition predicted nearly all of the dimensions of group cohesion at all time points using longitudinal analyses. We also found that social and task-based forms of communication had more consistent patterns of relationships with the social and task-based dimensions of group cohesion, supporting the hypothesis that different group-interaction variables would predict different dimensions of group cohesion. Contrary to our hypotheses, perceptions of cooperation did not demonstrate a consistent relationship with any dimension of group cohesion or across time-points.

One of the more interesting, and perhaps unexpected, findings was the degree to which friendly competition was consistently and positively related across group cohesion dimensions. There is evidence that when groups set a goal based upon the summing of individual progress it results in a perceived conjunctive task, where the success is based upon not only the expertise of the highest performing members, but is also limited by the progress of the lowest performing members [34,35]. In HIP, participants set a shared goal within their teams to achieve across the duration of the study. At the end of each intervention session, progress toward the team goal was reviewed and compared to other teams. Teams that met their goal were celebrated, fostering a sense of friendly competition between teams. This type of friendly competition has been highlighted as a possible motivating feature in a number of other studies with different populations. Ingram and colleagues provided qualitative data that highlighted the motivational aspects of measuring up to the standards of others in the group [36]. Further, Buis and colleagues hypothesized that the positive relationship between competition and physical activity goal completion found in their study was due to a sense of group accountability or cohesion [26]. Finally, in a group of African American men, Hooker and colleagues used a similar small, within-group competition to successfully increase physical activity [27]. The analyses showed that friendly competition predicted cohesion at each time point, indicating that friendly competition may be a key group-interaction for effective group-based interventions.

Perhaps counter-intuitively, people have been known to find greater attraction to their competitors rather than noncompetitors [37]. In the same way team members may compete with those who hold a similar position, ethnic minority women in the health education intervention may compare themselves to others in the group who share similar life-roles to them (e.g., group member, wife, mother, friend) [37]. Friendly competition was one group dynamics strategy in this intervention that increased a sense of belonging.

Perhaps a less abstract explanation for the potential role of friendly competition to predict group cohesion is simply that participants like to try to be the best in their own groups. For example, Green and colleagues [38] successfully harnessed this idea of friendly competition in their study of group dynamics-based physical activity promotion in worksites, where worksites had team-based competitions. Recognition for successful competition was acknowledged by group praise. This competition, feedback, and reward approach also resulted in significant increases in physical activity [38]. Even in the absence of providing specific competition related prizes within HIP, we still observed the positive effect of competition on cohesion.

Our findings around task-based communication and the task-based aspects of group cohesion should not be surprising given the use of a number of strategies that encouraged participants to engage in discussions about physical activity. During HIP intervention sessions, topics, such as setting challenging, yet attainable, goals, overcoming barriers to doing physical activity with practical solutions, and increasing social support to achieve physical activity goals, were discussed in the larger group. Women were tasked with continuing the discussion in their teams and completing a related worksheet as a team to increase task-based communication. Communication around the task at hand can be facilitated through mechanisms such as group problem-solving and has been used successfully in other studies [39,40]. Our findings contribute new information to this body of literature—that group interactions may not only result in applicable plans for participants to achieve a goal, but may also foster a sense of cohesion that can increase motivation toward achieving the goal [15].

It was surprising that cooperation was not a consistent predictor of group cohesion over the course of this study. Our initial belief was that cooperation would be strongly related to the task-based aspects of group cohesion because of the previous findings that control beliefs, often developed through vicarious learning and support, predicted task related group cohesion [13]. There are a number of possible explanations for this. First, the sessions may not have included activities that the participants considered cooperative. However, this seems unlikely given the significant increase in participant perceptions of cooperation over the course of the program and because cooperative activities are a consistently reported aspect of group-based programs for physical activity [18]. Second, it could be that communication and friendly competition account for the role of cooperation within a physical activity environment. We did not propose such an indirect relationship prior to completing our analyses, but suggest this may be an interesting area of future investigation.

This was the first study to determine the predictive relationship of group-interaction variables to group cohesion. The measures for group-interaction variables were developed specifically for this project, and, although they demonstrated internal consistency and predictive validity, further validity and reliability testing is warranted. In addition, the analyses were limited to participants who had both the baseline and follow-up assessments for group interaction and cohesion variables resulting in findings that cannot be generalized broadly. Nevertheless, the investigation of these relationships in a large minority population, over time provided the opportunity to speak more definitively about the consistent relationships that were found. In the same vein, the ethnic and racial composition of this sample may influence the generalizability of these results to other groups (e.g., mixed-race, mixed-gender).

These results help to decrease the paucity in the literature around the relationship between group-interaction variables and group cohesion. In a recent systematic review of group dynamics-based physical activity interventions it was concluded that more research is needed to determine what mechanisms lead to the robust effect of these interventions [18]. Group-interaction variables are a direct way in which to influence the perception of cohesion. Strategies that foster friendly competition will be the most likely to improve participant perceptions of group cohesion, and cooperation lacked a consistent pattern of prediction. Future research is also needed to expand upon this exploratory study to determine the degree to which group interaction and group cohesion mediate an increase in physical activity or program adherence.

Competition was a greater, and more consistent, predictor of cohesion over the other group-interaction variables. These data for ethnic minority women are of particular interest as females are seen as the more cooperative and collective gender [41,42] and as ethnic minority groups in America have been known to seek group identity and shared achievement [43]. It has been documented that competition is an influential factor for African American men engaging in physical activity interventions [44]. This is the first study, to our knowledge, to find such a strong relationship between competition and a sense of group cohesion in African American and Latino women. Reasons attributed to the appeal of competition for men (e.g., showing off, sense of accomplishment) provide new insights to the assumed cooperative female gender and suggest that interpersonal relationships that support positive health behavior changes are more complex than previously suggested. Last, the effects of competition are stronger for males than females in mixed-gender environments [45]. Future research endeavors are needed to see if these findings are generalizable to other all-female physical activity groups. If so, including elements of the competitive side of physical activity as a promotion strategy might help women to engage in and sustain physical activity.

## Conclusions

Group dynamics-based physical activity programs are successful at achieving their outcomes of interest [18]. Although previous research has shown that increased perceptions of cohesion lead to increased engagement in the program and subsequent increases in physical activity [4], less is known about what strategies lead to increased perceptions of cohesion [18]. This study presents promising data about how group-interaction variables, including communication, competition, and cooperation, may influence the perception of cohesion. Specific investigation of these variables indicated that strategies that foster friendly competition are most likely to improve participant perceptions of group cohesion.

## Abbreviations

HIP: Health is power; ATG-T: Attraction to the group’s task-based activities; ATG-S: Attraction to the group’s social activities; GI-T: Group integration around task activities; GI-S: Group’s integration around social activities.

## Competing interests

The author declares that they have no competing interests.

## Authors’ contributions

SMH ran the analyses and wrote the manuscript with the assistance of PAE, who also developed the measure being tested. REL and SKM developed and delivered the intervention and contributed to the draft of the manuscript. All authors contributed to the editing and approval of the final manuscript.

## Supplementary Material

Additional file 1 Complete group-interaction variables on a 9-point agreement scale. Click here for file
