# Supplementary material for: Longitudinal analysis of minority women’s perceptions of cohesion: the role of cooperation, communication, and competition
Source: Int J Behav Nutr Phys Act. 2014 Apr 29;11:57. doi: 10.1186/1479-5868-11-57 (PMC4108125; doi:10.1186/1479-5868-11-57)
Supplement: Additional file 1 — Complete group-interaction variables on a 9-point agreement scale. [file 1479-5868-11-57-S1.docx]

| **PART A** |  |  |  |  |  |  |  |  |  |  |
| --- | --- | --- | --- | --- | --- | --- | --- | --- | --- | --- |
| **Using the following scale, please bubble in a number from 1 to 9 to indicate your level of agreement with each of the statements.** | | | | | | | | | | |
| **If you neither agree nor disagree respond by using the number '5'.** | | | | | | | | | | |
|  |  |  |  |  |  |  |  |  |  |  |
|  |  | **Very Strongly Disagree** | **Strongly Disagree** | **Disagree** |  | **Neither Agree Nor Disagree** |  | **Agree** | **Strongly Agree** | **Very Strongly Agree** |
|  |  | **1** | **2** | **3** | **4** | **5** | **6** | **7** | **8** | **9** |
|  |  |  |  |  |  |  |  |  |  |  |
| COMPETITION | | |  |  |  |  |  |  |  |  |
| I try to do the same things the healthiest people of this group are doing. | | 🔾 | 🔾 | 🔾 | 🔾 | 🔾 | 🔾 | 🔾 | 🔾 | 🔾 |
|  |  |  |  |  |  |  |  |  |  |  |
| I would like to be the healthiest person of this group. | | 🔾 | 🔾 | 🔾 | 🔾 | 🔾 | 🔾 | 🔾 | 🔾 | 🔾 |
|  |  |  |  |  |  |  |  |  |  |  |
| There is friendly competition within the members to stay as healthy as possible. | | 🔾 | 🔾 | 🔾 | 🔾 | 🔾 | 🔾 | 🔾 | 🔾 | 🔾 |
|  |  |  |  |  |  |  |  |  |  |  |
| COMMUNICATION | | | |  |  |  |  |  |  |  |
| People of this group talk about things that are happening in our lives. | | 🔾 | 🔾 | 🔾 | 🔾 | 🔾 | 🔾 | 🔾 | 🔾 | 🔾 |
|  |  |  |  |  |  |  |  |  |  |  |
| People share stories about themselves in our class. | |  |  |  |  |  |  |  |  |  |
| Our group discusses the importance of regular physical activity. | | 🔾 | 🔾 | 🔾 | 🔾 | 🔾 | 🔾 | 🔾 | 🔾 | 🔾 |
|  |  |  |  |  |  |  |  |  |  |  |
| Members of our group talk about how often they should do physical activity. | | 🔾 | 🔾 | 🔾 | 🔾 | 🔾 | 🔾 | 🔾 | 🔾 | 🔾 |
|  |  |  |  |  |  |  |  |  |  |  |
| Members of our group discuss the appropriate type of physical activity we should do. | | 🔾 | 🔾 | 🔾 | 🔾 | 🔾 | 🔾 | 🔾 | 🔾 | 🔾 |
|  |  |  |  |  |  |  |  |  |  |  |
| Members of our group talk about exercise and physical activity a lot. | | 🔾 | 🔾 | 🔾 | 🔾 | 🔾 | 🔾 | 🔾 | 🔾 | 🔾 |
|  |  |  |  |  |  |  |  |  |  |  |
| COOPERATION | |  |  |  |  |  |  |  |  |  |
| 15. We all cooperate to help this group's program run smoothly. | | 🔾 | 🔾 | 🔾 | 🔾 | 🔾 | 🔾 | 🔾 | 🔾 | 🔾 |
|  |  |  |  |  |  |  |  |  |  |  |
| 16. If people want to do different things at the program we cooperate to satisfy everyone. | | 🔾 | 🔾 | 🔾 | 🔾 | 🔾 | 🔾 | 🔾 | 🔾 | 🔾 |
|  |  |  |  |  |  |  |  |  |  |  |
| 17. Members of our group cooperate well together. | | 🔾 | 🔾 | 🔾 | 🔾 | 🔾 | 🔾 | 🔾 | 🔾 | 🔾 |
